# A cross-sectional study of smoking and depression among US adults: NHANES (2005–2018)

**DOI:** 10.3389/fpubh.2023.1081706

**Published:** 2023-01-30

**Authors:** Zhaoping Wu, Qiang Yue, Zhen Zhao, Jun Wen, Lanying Tang, Zhenzhen Zhong, Jiahui Yang, Yingpu Yuan, Xiaobo Zhang

**Affiliations:** ^1^Department of Neurology, Zhuzhou Central Hospital, Zhuzhou, Hunan, China; ^2^Department of Neurology, First People's Hospital of Changde City, Changde, Hunan, China; ^3^Department of Critical Care Medicine, First People's Hospital of Changde City, Changde, Hunan, China

**Keywords:** smoking, smoking cessation, depression, NHANES, PHQ-9

## Abstract

**Background:**

The relationship between smoking and depression remains controversial. This study aimed to investigate the association between smoking and depression from three aspects: smoking status, smoking volume, and smoking cessation.

**Methods:**

Data from adults aged ≥20 who participated in the National Health and Nutrition Examination Survey (NHANES) between 2005 and 2018 were collected. The study gathered information about the participants' smoking status (never smokers, previous smokers, occasional smokers, daily smokers), smoking quantity per day, and smoking cessation. Depressive symptoms were assessed using the Patient Health Questionnaire (PHQ-9), with a score ≥10 indicating the presence of clinically relevant symptoms. Multivariable logistic regression was conducted to evaluate the association of smoking status, daily smoking volume, and smoking cessation duration with depression.

**Results:**

Previous smokers [odds ratio (OR) = 1.25, 95% confidence interval (CI): 1.05–1.48] and occasional smokers (OR = 1.84, 95% CI: 1.39–2.45) were associated with a higher risk of depression compared with never smokers. Daily smokers had the highest risk of depression (OR = 2.37, 95% CI: 2.05–2.75). In addition, a tendency toward a positive correlation was observed between daily smoking volume and depression (OR = 1.65, 95% CI: 1.24–2.19) (*P* for trend < 0.05). Furthermore, the longer the smoking cessation duration, the lower the risk of depression (OR = 0.55, 95% CI: 0.39–0.79) (*P* for trend < 0.05).

**Conclusions:**

Smoking is a behavior that increases the risk of depression. The higher the smoking frequency and smoking volume, the higher the risk of depression, whereas smoking cessation is associated with decreased risk of depression, and the longer the smoking cessation duration, the lower the risk of depression.

## 1. Introduction

Depression has become a major and serious public health challenge worldwide and has been recognized as one of the leading causes of disability and mortality around the globe (1–3). In the United States, the prevalence of major depressive disorders in women is as high as 21% and is more than 10% in men (4, 5). Thus, there is an urgent need to investigate the influencing and mitigating factors of depression in the US population. Despite growing evidence that smoking may be a risk factor for psychological problems, there has been controversy about the relationship between smoking and depression (6). A Mendelian randomization study of 462,690 participants from the UK Biobank showed that smoking was associated with an increased risk of depression and even schizophrenia in both lifelong and beginning smokers (1). In contrast, a systematic review of mental health professionals' attitudes toward smoking and smoking cessation in people with mental illness, including 38 small studies with 16,369 participants, found that smoking mitigated some psychological health problems, including depression, anxiety, and stress (7). Furthermore, some researchers did not find any association between persistent smoking and an increased risk of depression after controlling for confounding factors such as family or genetic susceptibility (8). Given these contradictions, a more in-depth study of the relationship between smoking and depression is necessary.

The change in depressive symptoms after smoking cessation is another aspect reflecting the relationship between smoking and depression, which too has attracted controversy. Unlike their counterparts in other countries, mental health professionals in the US do not consider the adverse effects of smoking cessation (7). Research has shown that smoking cessation could improve mental health (9), even in people with mental illness (6, 10), and found that continued smoking cessation over 10 years was associated with a lower risk of major depression (4). In contrast, some studies report that smoking reduces stress and relieves anxiety, and quitting smoking can lead to the aggravation of mental symptoms (11). Inconsistent findings on the relationship between smoking cessation and depressive symptoms highlight the need for continued research to explore this issue.

This study aimed to examine the association between smoking and depressive symptoms in a large, nationally representative sample of US adults across multiple dimensions by adjusting for various potential confounders. Specifically, we focused on analyzing the association between smoking and depression from three aspects: smoking status, smoking volume, and smoking cessation.

## 2. Methods

### 2.1. Study population

**Figure 1:**
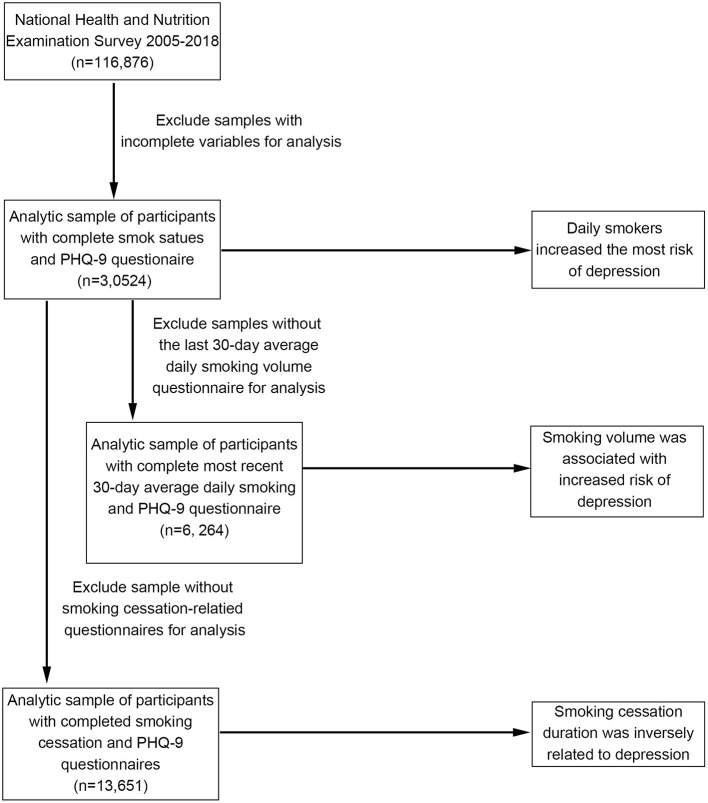
Flowchart of the sample selection from NHANES (2005–2018).

National Health and Nutrition Examination Survey (NHANES) is a nationally representative, complex, stratified, multistage, cross-sectional survey to investigate the risk factors associated with health and nutrition among the US population. Since 1999, NHANES has been conducted biennially, and each survey round includes different participants. The assessment is carried out by means of home interviews and mobile medical examination centers (12). The National Center for Health Statistics Research Ethics Review Board approves the survey, and all participants provide written informed consent. Our study used data from seven rounds of NHANES between 2005 and 2018. This period was focused on because the NHANES started using the Patient Health Questionnaire (PHQ-9) score to assess depression in 2005, and the COVID-19 pandemic had not yet hit the world in 2018. Samples with incomplete questionnaire information in the seven survey rounds were excluded from this study. Considering that the sampling age of most participants was greater than or equal to 20, we chose people aged ≥20 as the research subjects. Figure 1 summarizes the inclusion and exclusion criteria for each aspect of the study.

### 2.2. Exposure: Smoking

Based on the answer to the question “Have you smoked at least 100 cigarettes in life,” we defined the participants who answered “no” as “never smokers.” Those who answered “yes” were further divided into three groups according to the answer to the question “Do you now smoke cigarettes?”: “previous smokers,” “occasional smokers,” and “daily smokers.” The question “On average, how many cigarettes have you smoked per day during the past 30 days?” examined smoking volume. The average number of cigarettes smoked per day by an individual was coded as the smoking volume; the individuals who responded with “1 cigarette or less per day” were coded as 1, and those who answered “95 cigarettes or more per day” were coded as 95. The above data were treated as a continuous variable analysis. The participants were divided into quartile groups according to the smoking volume from low to high: <5 cigarettes per day (Q1), 5–9 cigarettes per day (Q2), 10–19 cigarettes per day (Q3), and more than 20 cigarettes per day (Q4).

The question “How long has it been since you quit smoking cigarettes” obtained data on smoking cessation. Smoking cessation was computed in years. Smoking cessation duration was considered “0” (Q0) when participants responded to “Do you now smoke cigarettes” with “occasionally” or “daily.” The participants were divided into quartile groups according to the duration of smoking cessation from low to high: <5 years (Q1), 5–15 years (Q2), 15–29 years (Q3), and more than 29 years (Q4).

### 2.3. Covariates

Potential confounders for depression were selected based on previous studies, such as gender, age, race, education level, marital status, family poverty income ratio (PIR), body mass index (BMI), diabetes, failing kidneys, heart failure, coronary heart disease, emphysema, chronic bronchitis, and cancer or malignancy (13). Race was self-reported, and participants were categorized into Mexican American, non-Hispanic black, non-Hispanic white, Hispanic, and other races. Marital status was categorized into married, living with a partner, divorced, widowed or separated, and never married. Education level was divided into several subgroups (college graduates or above, associate (AA) degree, high school graduates, and below grade 11) based on the “Education level—Adults 20+” questionnaire. Family PIR was expressed as median household income divided by the poverty threshold (14). Diabetes status in adults was coded as yes, no, or borderline.

### 2.4. Statistical analysis

All statistical analyses were performed using R software version 4.2.0 and the nhanesR package, a survey analysis procedure that accounts for sample weights, stratification, and clustering of complex sampling designs to ensure nationally representative estimates. Continuous variables in baseline data were presented as weighted means and standard errors; categorical variables were presented as weighted percentages and frequencies. Differences between continuous variables were assessed using the analysis of variance (ANOVA) test or the Kruskal–Wallis test, depending on the distribution. Differences between categorical variables were analyzed using the chi-square test. Three logistic regression models were developed to express the association of smoking status, smoking volume, and smoking cessation with depression. In Model I, no adjustment was made for confounders. In Model II, age, gender, BMI, and race were adjusted based on Model I. In Model III, adjustments were made for education level, marital status, family PIR, diabetes, failing kidneys, heart failure, coronary heart disease, emphysema, chronic bronchitis, and cancer or malignancy based on Model II. Two-sided values of *P* < 0.05 were considered statistically significant.

## 3. Results

### 3.1. Smoking status

A total of 116,876 US residents participated in seven survey rounds between 2008 and 2015. Of these, 43,190 participants provided complete data on smoking status and depressive symptoms; 12,666 participants were excluded due to missing covariate data. A total of 30,524 participants were included for the final analysis of the association between smoking status and depressive symptoms. Their mean age was 47.17 ± 0.24 years, and 48.70% were male.

Among the analyzed sample participants, 54.8% (*n* = 16,724) were never smokers, 24.5% (*n* = 7,479) were previous smokers, 3.8% (*n* = 1,160) were occasional smokers, and 16.9% (*n* = 5,161) were daily smokers. Compared with other groups, daily smokers were younger, primarily men, non-Hispanic whites and non-Hispanic blacks, and had lower BMI, family PIR, and education levels. In addition, they were most likely to be divorced or living with their parents, suffering from heart failure, emphysema, chronic bronchitis, depression, and other diseases. The specific baseline characteristics are shown in Table 1.

**Table 1:** Baseline characteristics of participants with smoking status (*n* = 30,524).

**Characteristics**	**Smoking status**					***P***
	**Total** **(*****n*** = **30,524)**	**Never smokers** **(*****n*** = **16,724, 54.8%)**	**Previous smokers** **(*****n*** = **7,479, 24.5%)**	**Occasional smokers** **(*****n*** = **1,160, 3.8%)**	**Daily smokers** **(*****n*** = **5,161, 16.9%)**	
Age, year (mean, se)	47.17 ± 0.24	45.65 ± 0.28	53.83 ± 0.35	38.74 ± 0.56	43.62 ± 0.29	< 0.001
BMI, kg/m^2^ (mean, se)	29.10 ± 0.08	29.13 ± 0.10	29.83 ± 0.13	28.92 ± 0.30	27.93 ± 0.12	< 0.001
Family PIR (mean, se)	3.03 ± 0.03	3.19 ± 0.04	3.22 ± 0.04	2.68 ± 0.08	2.30 ± 0.04	< 0.001
**Gender (** ***n*** **, %)**						
Male	14,947 (48.70%)	6,820 (42.78%)	4,520 (57.15%)	716 (59.44%)	2,891 (53.20%)	< 0.001
Female	15,577 (51.30%)	9,904 (57.22%)	2,959 (42.85%)	444 (40.56%)	2,270 (46.80%)	
**Race (** ***n*** **, %)**						
Mexican American	4,593 (7.96%)	2,891 (9.34%)	1,014 (6.25%)	259 (15.41%)	429 (4.35%)	< 0.001
Other Hispanic	2,750 (5.11%)	663 (5.88%)	657 (4.23%)	106 (6.23%)	324 (3.63%)	
Non-Hispanic white	13,491 (69.08%)	6,353 (64.85%)	4,020 (77.78%)	381 (56.20%)	2,737 (72.75%)	
Non-Hispanic black	6,494 (10.74%)	3,665 (11.76%)	1,230 (6.60%)	303 (14.14%)	1,296 (12.85%)	
Other race	3,196 (7.12%)	2,152 (8.17%)	558 (5.14%)	111 (8.03%)	375 (6.41%)	
**Education level (** ***n*** **, %)**						
< 9th grade	2,829 (4.65%)	1,550 (4.38%)	788 (5.07%)	97 (4.44%)	394 (4.95%)	< 0.001
9–11th grade	4,231 (10.17%)	1,794 (7.18%)	994 (9.71%)	210 (12.98%)	1,233 (20.11%)	
High school graduate	7,060 (23.27%)	3,404 (19.84%)	1,751 (23.82%)	287 (24.86%)	1,618 (33.42%)	
Some college or AA degree	9,212 (31.82%)	5,034 (31.17%)	2,295 (32.84%)	367 (33.43%)	1,516 (32.11%)	
College graduate or above	7,192 (30.09%)	4,942 (37.43%)	1,651 (28.55%)	199 (24.29%)	400 (9.41%)	
**Marital status (** ***n*** **, %)**						
Married	15,852 (55.85%)	9,094 (58.64%)	4,415 (62.59%)	455 (41.19%)	1,888 (39.67%)	< 0.001
Widowed	2,275 (5.39%)	1,211 (5.17%)	741 (7.06%)	43 (1.98%)	280 (4.35%)	
Divorced	3,390 (10.43%)	1,469 (8.43%)	953 (11.71%)	134 (10.37%)	834 (15.14%)	
Separated	1,006 (2.35%)	470 (1.89%)	206 (1.83%)	50 (3.40%)	280 (4.43%)	
Never married	5,474 (17.75%)	3,317 (19.42%)	717 (10.31%)	336 (30.02%)	1,104 (20.76%)	
Living with partner	2,527 (8.23%)	1,163 (6.46%)	447 (6.50%)	142 (13.04%)	775 (15.65%)	
**Diabetes (** ***n*** **, %)**						
Yes	3,837 (9.36%)	1,904 (8.42%)	1,341 (13.31%)	82 (4.06%)	510 (7.69%)	< 0.001
Borderline	708 (2.13%)	348 (1.98%)	238 (2.83%)	24 (1.39%)	98 (1.75%)	
No	25,979 (88.51%)	14,472 (89.60%)	5,900 (83.86%)	1,054 (94.55%)	4,553 (90.56%)	
**Failing kidneys (** ***n*** **, %)**						
Yes	930 (2.37%)	419 (1.98%)	351 (3.42%)	21 (1.70%)	139 (2.26%)	< 0.001
No	29,594 (97.63%)	16,305 (98.02%)	7,128 (96.58%)	1,139 (98.30%)	5,022 (97.74%)	
**Heart failure (** ***n*** **, %)**						
Yes	928 (2.20%)	364 (1.53%)	383 (3.65%)	27 (1.73%)	154 (2.32%)	< 0.001
No	29,596 (97.80%)	16,360 (98.47%)	7,096 (96.35%)	1,133 (98.27%)	5,007 (97.68%)	
**Coronary heart disease (** ***n*** **, %)**						
Yes	1,191 (3.28%)	440 (2.16%)	542 (5.95%)	27 (2.59%)	182 (3.08%)	< 0.001
No	29,333 (96.72%)	16,284 (97.84%)	6,937 (94.05%)	1,133 (97.41%)	4,979 (96.92%)	
**Emphysema (** ***n*** **, %)**						
Yes	620 (1.76%)	47 (0.23%)	297 (3.32%)	18 (1.55%)	258 (4.53%)	< 0.001
No	29,904 (98.24%)	16,677 (99.77%)	7,182 (96.68%)	1,142 (98.45%)	4,903 (95.47%)	
**Chronic bronchitis (** ***n*** **, %)**						
Yes	1,799 (5.95%)	118 (3.79%)	180 (7.18%)	5,234 (5.16%)	88 (11.41%)	< 0.001
No	28,725 (94.05%)	16,110 (96.21%)	6,916 (92.82%)	1,102 (94.84%)	4,597 (88.59%)	
**Cancer or malignancy (** ***n*** **, %)**						
Yes	2,920 (10.07%)	1,309 (8.45%)	1,151 (15.32%)	62 (6.14%)	398 (8.39%)	< 0.001
No	27,604 (89.93%)	15,415 (91.55%)	6,328 (84.68%)	1,098 (93.86%)	4,763 (91.61%)	
**Depression (** ***n*** **, %)**						
Yes	2,614 (7.58%)	1,049 (5.34%)	583 (6.74%)	128 (10.41%)	854 (15.61%)	< 0.001
No	27,910 (92.42%)	15,675 (94.66%)	6,896 (93.26%)	1,032 (89.59%)	4,307 (84.39%)	

Continuous variables are mean and variance, and categorical variables are proportions and confidence intervals.

We observed significant differences in gender, BMI, race, education level, marital status, and family PIR between depressed and non-depressed individuals (*P* < 0.05) (Table 2). Diabetes, renal failure, heart failure, coronary heart disease, chronic bronchitis, emphysema, nausea, and tumor were highly correlated with depression (*P* < 0.05) (Table 2). Furthermore, daily smokers had the strongest association with depression compared with the other three groups (OR = 3.28, 95% CI: 2.90–3.71, *P* < 0.001).

**Table 2:** Univariate analysis of depressive symptoms in the study population based on smoking status.

**Characteristics**	**Statistics**	**OR, 95%CI, *P*-value**
Age, year (mean, se)	47.17 ± 0.24	1.00 (0.99–1.00) 0.100
BMI, kg/m^2^ (mean, se)	29.10 ± 0.08	1.03 (1.02–1.04) < 0.001
Family PIR (mean, se)	3.03 ± 0.03	0.68 (0.65–0.71) < < 0.001
**Gender (** ***n*** **, %)**		
Male	14,947 (48.70%)	1
Female	15,577 (51.30%)	1.81 (1.63–2.01) < 0.001
**Race (** ***n*** **, %)**		
Mexican American	4,593 (7.96%)	1
Other Hispanic	2,750 (5.11%)	1.49 (1.20–1.85) < 0.001
Non-Hispanic white	13,491 (69.08%)	1.00 (0.84–1.19) 0.997
Non-Hispanic black	6,494 (10.74%)	1.34 (1.14–1.58) < 0.001
Other races	3,196 (7.12%)	1.05 (0.84–1.30) 0.689
**Education level (** ***n*** **, %)**		
< 9th grade	2,829 (4.65%)	1
9–11th grade	4,231 (10.17%)	1.04 (0.88–1.22) 0.645
High school graduate/GED or equivalent	7,060 (23.27%)	0.71 (0.60–0.83) < 0.001
Some college or AA degree	9,212 (31.82%)	0.64 (0.54–0.77) < 0.001
College graduate or above	7,192 (30.09%)	0.27 (0.21–0.35) < 0.001
**Marital status (** ***n*** **, %)**		
Married	15,852 (55.85%)	1
Widowed	2,275 (5.39%)	2.19 (1.80–2.65) < 0.001
Divorced	3,390 (10.43%)	2.78 (2.42–3.20) < 0.001
Separated	1,006 (2.35%)	3.92 (3.26–4.72) < 0.001
Never married	5,474 (17.75%)	1.88 (1.64–2.16) < 0.001
Living with partner	2,527 (8.23%)	2.15 (1.78–2.59) < 0.001
**Diabetes (** ***n*** **, %)**		
No	25,979 (88.51%)	1
Borderline	708 (2.13%)	1.65 (1.15–2.36) 0.008
Yes	3,837 (9.36%)	1.84 (1.60–2.11) < 0.001
**Failing kidneys (** ***n*** **, %)**		
No	29,594 (97.63%)	1
Yes	930 (2.37%)	3.13 (2.57–3.80) < 0.001
**Heart failure (** ***n*** **, %)**		
No	29,596 (97.80%)	1
Yes	928 (2.20%)	2.80 (2.27–3.46) < 0.001
**Coronary heart disease (** ***n*** **, %)**		
No	29,333 (96.72%)	1
Yes	1,191 (3.28%)	1.73 (1.35–2.20) < 0.001
**Emphysema (** ***n*** **, %)**		
No	29,904 (98.24%)	1
Yes	620 (1.76%)	3.29 (2.63–4.13) < 0.001
**Chronic bronchitis (** ***n*** **, %)**		
No	28,725 (94.05%)	1
Yes	1,799 (5.95%)	3.31 (2.85–3.84) < 0.001
**Cancer or malignancy (** ***n*** **, %)**		
No	27,604 (89.93%)	1
Yes	2,920 (10.07%)	1.17 (0.98–1.41) 0.091
**Smoke status**		
Never smokers	16,724 (54.8%)	1
Previous smokers	7,479 (24.5%)	1.28 (1.09–1.50) 0.003
Occasional smokers	1,160 (3.8%)	2.06 (1.58–2.69) < 0.001
Daily smokers	5,161 (16.9%)	3.28 (2.90–3.71) < 0.001

CI, confidence interval; OR, odds ratio. Continuous variables are mean and variance, and categorical variables are proportions and confidence intervals.

Table 3 shows that the association between smoking status and depression persisted after multiple adjustments for different confounders. Compared with never smokers, participants in the other three groups had an increased risk of depression (all models, *P* for trend < 0.001). Furthermore, compared with previous smokers (OR = 1.25, 95% CI: 1.05–1.48, *P* < 0.05) and occasional smokers (OR = 1.84, 95% CI: 1.39–2.45, *P* < 0.05), daily smokers had a 1.3–1.9-fold increased risk of depression (OR = 2.37, 95% CI: 2.05–2.75, *P* < 0.05).

**Table 3:** Associations of smoking status with clinically relevant depressive symptoms among adults (*n* = 16,724) in NHANES (2005–2018).

**Exposure**	**OR (95%CI)**, ***P*****-value**		
	**Model 1**	**Model 2**	**Model 3**
**Smoking status**			
Never smokers	1	1	1
Previous smokers	1.28 (1.09–1.50) 0.003	1.43 (1.21–1.68) < 0.001	1.25 (1.05–1.48) 0.013
Occasional smokers	2.06 (1.58–2.69) < 0.001	2.30 (1.74–3.05) < 0.001	1.84 (1.39–2.45) < 0.001
Daily smokers	3.28 (2.90–3.71) < 0.001	3.82 (3.35–4.36) < 0.001	2.37 (2.05–2.75) < 0.001
*P* for trend	< 0.001	< 0.001	< 0.001

Model 1: adjust for none. Model 2: adjust for age, gender, BMI, race. Model 3: adjust for age, gender, BMI, race, educational level, marital status, family PIR, diabetes, failing kidneys, heart failure, coronary heart disease, emphysema, chronic bronchitis, cancer or malignancy.

P-values for trends were calculated by modeling rank values for smoking status as continuous variables.

CI, confidence interval; OR, odds ratio.

### 3.2. Smoking volume

Among the participants, 6,246 provided complete daily smoking data for the past 30 days. Their average age was 42.74 ± 0.27 years, and 48.70% were male. Based on baseline results for average daily smoking in the four groups (Q1 to Q4), compared to the other three groups, the Q4 group that smoked the most number of cigarettes per day was older with fewer females, and mainly included non-Hispanic whites, widowed or divorced people, who had lower education level and higher prevalence of diabetes, emphysema, chronic bronchitis, cancer or malignant tumors, depression, and other diseases (Table 4).

**Table 4:** Baseline characteristics of participants with average daily smoking in the past 30 days.

**Characteristics**	**Average number of cigarettes smoked per day in the past 30 days**					***P***
	**Total** **(*****n*** = **6,264)**	**Q1** **(*****n*** = **1,475)**	**Q2** **(*****n*** = **1,085)**	**Q3** **(*****n*** = **1,869)**	**Q4** **(*****n*** = **1,835)**	
Age, year (mean, se)	42.74 ± 0.27	39.27 ± 0.51	40.19 ± 0.60	43.22 ± 0.46	45.57 ± 0.44	< 0.001
BMI, kg/m^2^ (mean, se)	28.10 ± 0.11	28.62 ± 0.27	28.26 ± 0.26	27.69 ± 0.18	28.09 ± 0.21	0.020
Family PIR (mean, se)	2.37 ± 0.04	2.39 ± 0.07	2.37 ± 0.07	2.40 ± 0.05	2.33 ± 0.06	0.790
**Gender (** ***n*** **, %)**						
Male	3,576 (54.34%)	859 (57.56%)	574 (48.70%)	1,030 (49.96%)	1,113 (58.89%)	< 0.001
Female	2,688 (45.66%)	616 (42.44%)	511 (51.30%)	839 (50.04%)	722 (41.11%)	
**Race (** ***n*** **, %)**						
Mexican American	668 (6.13%)	322 (14.95%)	138 (8.74%)	135 (3.88%)	73 (1.65%)	< 0.001
Other Hispanic	1,588 (13.09%)	426 (17.71%)	425 (23.11%)	486 (12.91%)	251 (5.91%)	
Non-Hispanic white	3,108 (70.10%)	424 (50.81%)	365 (56.73%)	986 (72.64%)	1,333 (85.53%)	
Non-Hispanic black	423 (4.07%)	164 (7.56%)	78 (5.17%)	109 (3.44%)	72 (2.02%)	
Other races	477 (6.61%)	139 (8.96%)	79 (6.25%)	153 (7.13%)	106 (4.90%)	
**Education level (** ***n*** **, %)**						
< 9th grade	484 (4.83%)	161 (6.39%)	79 (4.05%)	99 (3.29%)	145 (5.62)	< 0.001
9–11th grade	1,433 (18.89%)	308 (16.06%)	221 (16.13%)	438 (19.34%)	466 (21.44%)	
High school graduate	1,890 (31.92%)	361 (25.61%)	323 (29.52%)	588 (32.18%)	618 (36.59%)	
Some college or AA degree	1,871 (32.39%)	451 (32.55%)	353 (36.79%)	573 (33.46%)	494 (29.35%)	
College graduate or above	586 (11.97%)	194 (19.39%)	109 (13.52%)	171 (11.73%)	112 (7.00%)	
**Marital status (** ***n*** **, %)**						
Married	2,312 (39.81%)	532 (38.83%)	376 (35.87%)	655 (37.85%)	749 (43.94)	< 0.001
Widowed	321 (3.94%)	63 (3.06%)	46 (2.98%)	116 (5.16%)	96 (3.80%)	
Divorced	964 (14.33%)	185 (10.01%)	143 (12.75%)	308 (15.70%)	328 (16.45%)	
Separated	327 (4.25%)	77 (3.91%)	59 (4.69%)	97 (3.79%)	94 (4.67%)	
Never married	1,430 (22.46%)	413 (29.15%)	292 (26.27%)	403 (20.91%)	322 (18.08%)	
Living with partner	910 (15.21%)	205 (15.05%)	169 (17.43%)	290 (16.59%)	246 (13.06%)	
**Diabetes (** ***n*** **, %)**						
No	5,554 (91.24%)	1,321 (92.80%)	966 (91.70%)	1,677 (92.08%)	1,590 (89.34%)	0.030
Borderline	121 (1.68%)	31 (1.65%)	30 (2.03%)	28 (1.55%)	32 (1.67%)	
Yes	589 (7.07%)	123 (5.54%)	89 (6.27%)	164 (6.37%)	213 (8.99%)	
**Failing kidneys (** ***n*** **, %)**						
Yes	160 (2.17%)	32 (2.04%)	29 (2.12%)	43 (1.87%)	56 (2.55%)	0.580
No	6,104 (97.83%)	1,443 (97.96%)	1,056 (97.88%)	1,826 (98.13%)	1,779 (97.45%)	
**Heart failure (** ***n*** **, %)**						
Yes	179 (2.22%)	38 (2.30%)	30 (2.01%)	50 (2.07%)	61 (2.40%)	0.880
No	6,085 (97.78%)	1,437 (97.70%)	1,055 (97.99%)	1,819 (97.93%)	1,774 (97.60%)	
**Coronary heart disease (** ***n*** **, %)**						
Yes	203 (2.95%)	38 (2.57%)	22 (1.62%)	58 (3.28%)	85 (3.49%)	0.080
No	6,061 (97.05%)	1,437 (97.43%)	1,063 (98.38%)	1,811 (96.72%)	1,750 (96.51%)	
**Emphysema (** ***n*** **, %)**						
Yes	276 (4.03%)	26 (1.73%)	24 (1.80%)	80 (3.78%)	146 (6.65%)	< 0.001
No	5,988 (95.97%)	1,449 (98.27%)	1,061 (98.20%)	1,789 (96.22%)	1,689 (93.35%)	
**Chronic bronchitis (** ***n*** **, %)**						
Yes	620 (10.36%)	82 (6.02%)	82 (9.43%)	202 (10.30%)	254 (13.45%)	< 0.001
No	5,644 (89.64%)	1,393 (93.98%)	1,003 (90.57%)	1,667 (89.70%)	1,581 (86.55%)	
**Cancer or malignancy (** ***n*** **, %)**						
Yes	456 (7.96%)	83 (6.16%)	60 (5.99%)	136 (8.17%)	177 (9.76%)	0.010
No	5,808 (92.04%)	1,392 (93.84%)	1,025 (94.01%)	1,733 (91.83%)	1,658 (90.24%)	
**Depression (** ***n*** **, %)**						
Yes	976 (14.70%)	176 (11.33%)	150 (12.48%)	286 (15.02%)	364 (17.47%)	< 0.001
No	5,288 (85.30%)	1,299 (88.67%)	935 (87.52%)	1,583 (84.98%)	1,471 (82.53%)	

Continuous variables are mean and variance, and categorical variables are proportions and confidence intervals.

Q1, 1st quartile smoking volume (< 5 cigarettes per day); Q2, 2nd quartile smoking volume (5–9 cigarettes per day); Q3, 3rd quartile smoking volume (10–19 cigarettes per day); Q4, 4th quartile smoking volume (over 19 cigarettes per day).

Table 5 shows that BMI, race, education level, marital status, family PIR, hemoglobin A1c (HbA1c), and dietary intake significantly varied between depressed and non-depressed groups. Diabetes, renal failure, heart failure, coronary heart disease, chronic bronchitis, emphysema, nausea, and tumor were highly correlated with depression (*P* < 0.05). Compared with the other three groups, the Q4 group showed a higher correlation with depression (OR = 1.66, 95% CI: 1.29–2.12, *P* < 0.001).

**Table 5:** Univariate analysis of depressive symptoms in the study population based on average daily smoking in the past 30 days.

**Characteristics**	**Statistics**	**OR, 95%CI, *P*-value**
Age, year	42.74 ± 0.27	1.00 (0.99–1.01) 0.731
BMI, kg/m^2^	28.10 ± 0.11	1.02 (1.01–1.04) 0.002
Family PIR	2.37 ± 0.04	0.70 (0.65–0.75) < 0.001
**Gender (** ***n*** **, %)**		
Male	3,576 (54.34%)	1
Female	2,688 (45.66%)	2.31 (1.96–2.71) < 0.001
**Race (** ***n*** **, %)**		
Mexican American	668 (6.13%)	1
Other Hispanic	1,588 (13.09%)	1.60 (1.10–2.32) 0.015
Non-Hispanic white	3,108 (70.10%)	1.15 (0.87–1.51) 0.336
Non-Hispanic black	423 (4.07%)	1.23 (0.93–1.62) 0.150
Other races	477 (6.61%)	1.38 (0.97–1.96) 0.079
**Education level (** ***n*** **, %)**		
< 9th grade	484 (4.83%)	1
9–11th grade	1,433 (18.89%)	0.80 (0.61–1.06) 0.118
High school graduate	1,890 (31.92%)	0.57 (0.44–0.74) < 0.001
Some college or AA degree	1,871 (32.39%)	0.57 (0.43–0.76) < 0.001
College graduate or above	586 (11.97%)	0.27 (0.17–0.41) < 0.001
**Marital status (** ***n*** **, %)**		
Married	2,312 (39.81%)	1
Widowed	321 (3.94%)	2.35 (1.53–3.60) < 0.001
Divorced	964 (14.33%)	2.04 (1.60–2.61) < 0.001
Separated	327 (4.25%)	3.15 (2.44–4.08) < 0.001
Never married	1,430 (22.46%)	1.53 (1.21–1.93) < 0.001
Living with partner	910 (15.21%)	1.43 (1.11–1.84) 0.007
**Diabetes (** ***n*** **, %)**		
No	5,554 (91.24%)	1
Borderline	121 (1.68%)	1.05 (0.60–1.83) 0.876
Yes	589 (7.07%)	1.72 (1.33–2.22) < 0.001
**Failing kidneys (** ***n*** **, %)**		
No	6,104 (97.83%)	1
Yes	160 (2.17%)	4.01 (2.64–6.07) < 0.001
**Heart failure (** ***n*** **, %)**		
No	6,085 (97.78%)	1
Yes	179 (2.22%)	2.15 (1.41–3.27) < 0.001
**Coronary heart disease (** ***n*** **, %)**		
No	6,061 (97.05%)	1
Yes	203 (2.95%)	1.75 (1.15–2.66) 0.011
**Emphysema (** ***n*** **, %)**		
No	5,988 (95.97%)	1
Yes	276 (4.03%)	2.84 (2.09–3.85) < 0.001
**Chronic bronchitis (** ***n*** **, %)**		
No	5,644 (89.64%)	1
Yes	620 (10.36%)	2.76 (2.16–3.53) < 0.001
**Cancer or malignancy (** ***n*** **, %)**		
No	5,808 (92.04%)	1
Yes	456 (7.96%)	1.71 (1.27–2.29) < 0.001
**Average daily smoking in the past 30 days**		
Q1	1,475 (23.5%)	1
Q2	1,085 (17.3%)	1.12 (0.80–1.55) 0.515
Q3	1,869 (29.8%)	1.38 (1.04–1.85)0.030
Q4	1,835 (29.3%)	1.66 (1.29–2.12) < 0.001

CI, confidence interval; OR, odds ratio.

Continuous variables are mean and variance, and categorical variables are proportions and confidence intervals.

Q1, 1st quartile smoking volume (< 5 cigarettes per day); Q2, 2nd quartile smoking volume (5–9 cigarettes per day); Q3, 3rd quartile smoking volume (10–19 cigarettes per day); Q4, 4th quartile smoking volume (over 19 cigarettes per day).

**Figure 2:**
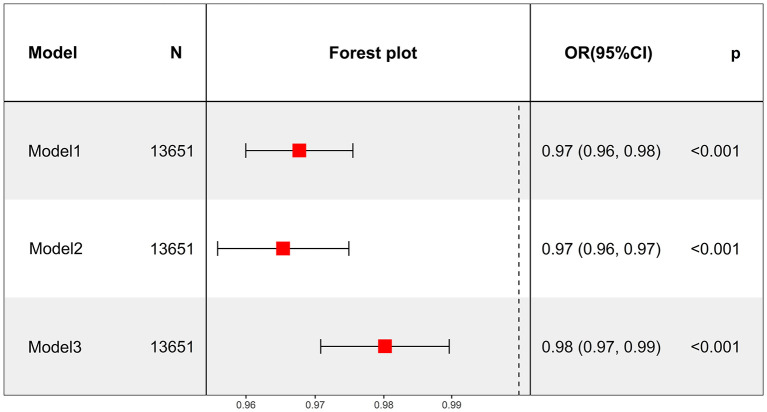
Average daily smoking in the past 30 days as a continuous variable to analyze the relationship between average daily smoking and depression.

The relationship between average daily smoking volume and depression was described by adjusting for different confounding factors. All three models showed that daily smoking was associated with an increased risk of depression. Model III showed that smoking one more cigarette a day was associated with a 2% increased likelihood of depression (OR = 1.02, 95% CI: 1.01–1.03, *P* < 0.001) (Figure 2). After converting continuous variables to categorical variables, all three models still showed that smoking was associated with an increased risk of depression (all models, *P* for trend < 0.001). Model III showed that compared with the Q1 group with the least daily smoking volume, the Q2 and Q3 groups had no significantly increased risk of depression (*P* > 0.05), whereas the Q4 group had a significantly increased risk of depression (OR = 1.65, 95% CI: 1.24–2.19, *P* < 0.001) (Table 6).

**Table 6:** Average daily smoking in the past 30 days as categorical variables to analyze the relationship between average daily smoking and depression in adults (*n* = 6,264) in NHANES (2005–2018).

	**OR (95%CI)**, ***P*****-value**		
	**Model 1**	**Model 2**	**Model 3**
**Average daily smoking in the past 30 days**			
Q1	1	1	1
Q2	1.12 (0.80–1.55) 0.515	1.08 (0.78–1.49) 0.637	1.05 (0.74–1.48) 0.784
Q3	1.38 (1.04–1.85) 0.030	1.46 (1.09–1.95) 0.012	1.36 (1.00–1.84) 0.054
Q4	1.66 (1.29–2.12) < 0.001	1.98 (1.51–2.60) < 0.001	1.65 (1.24–2.19) < 0.001
*P* for trend	< 0.001	< 0.001	< 0.001

Model 1: adjust for none. Model 2: adjust for age, gender, BMI, race; Model 3: adjust for age, gender, BMI, race, educational level, marital status, family PIR, diabetes, failing kidneys, heart failure, coronary heart disease, emphysema, chronic bronchitis, cancer or malignancy.

P for trend was calculated by modeling the median value of average number of cigarettes smoked per day in each quartile as a continuous variable.

CI, confidence interval; OR, odds ratio.

Q1, 1st quartile smoking volume (< 5 cigarettes per day); Q2, 2nd quartile smoking volume (5–9 cigarettes per day); Q3, 3rd quartile smoking volume (10–19 cigarettes per day); Q4, 4th quartile smoking volume (over 19 cigarettes per day).

### 3.3. Smoking cessation duration

A total of 13,651 people were examined in this analysis, including 7,330 quitters and 6,321 current smokers. Their mean age was 48.65 ± 0.27 years, and 55.82% were male. Table 7 shows that, compared with the other four groups, the Q4 group with the longest smoking cessation duration was older with higher income, married; included mostly males, non-Hispanic whites; and had higher education levels, a higher prevalence of diabetes, renal failure, heart failure, coronary heart disease, emphysema, chronic bronchitis, and cancer or malignant tumor.

**Table 7:** Baseline characteristics of participants with smoking cessation.

**Characteristics**	**Smoking cessation duration**						***P***
	**Total** **(*****n*** = **13,651)**	**Q0** **(*****n*** = **6,321)**	**Q1** **(*****n*** = **1,965)**	**Q2** **(*****n*** = **1,861)**	**Q3** **(*****n*** = **1,707)**	**Q4** **(*****n*** = **1,797)**	
Age, year (mean, se)	48.65 ± 0.27	42.76 ± 0.27	41.16 ± 0.47	50.39 ± 0.40	58.81 ± 0.40	67.55 ± 0.32	< 0.001
BMI, kg/m^2^ (mean, se)	29.06 ± 0.09	28.10 ± 0.11	29.70 ± 0.23	29.92 ± 0.21	30.14 ± 0.23	29.67 ± 0.23	< 0.001
Family PIR (mean, se)	2.84 ± 0.03	2.37 ± 0.04	2.95 ± 0.06	3.19 ± 0.05	3.42 ± 0.06	3.43 ± 0.06	< 0.001
**Gender (** ***n*** **, %)**							
Male	8,019 (55.80%)	3,607 (54.31%)	1,119 (57.43%)	1,082 (55.29%)	1,024 (56.22%)	1,187 (56.22%)	0.080
Female	5,632 (44.20%)	2,714 (45.69%)	846 (42.57%)	779 (44.71%)	683 (43.78%)	610 (43.78%)	
**Race (** ***n*** **, %)**							
Mexican American	1,691 (6.30%)	688 (6.32%)	333 (8.94%)	280 (7.02%)	217 (5.24%)	173 (3.09%)	< 0.001
Other Hispanic	1,080 (4.18%)	430 (4.09%)	186 (5.62%)	164 (4.25%)	160 (4.00%)	140 (2.79%)	
Non-Hispanic white	7,028 (74.12%)	3,118 (69.81%)	954 (72.54%)	936 (75.62%)	897 (78.73%)	1,123 (85.54%)	
Non-Hispanic black	2,820 (9.55%)	1,599 (13.08%)	318 (6.98%)	319 (6.96%)	316 (7.25%)	268 (5.28%)	
Other races	1,032 (5.84%)	486 (6.70%)	174 (5.93%)	162 (6.14%)	117 (4.77%)	93 (3.30%)	
**Education level (** ***n*** **, %)**							
< 9th grade	1,260 (4.95%)	491 (4.86%)	169 (4.20%)	199 (5.07%)	180 (5.19%)	221 (5.8)	< 0.001
9–11th grade	2,417 (13.80%)	1,443 (18.84%)	276 (10.31%)	277 (10.80%)	231 (9.69%)	190 (7.45%)	
High school graduate	3,621 (27.49%)	1,905 (31.89%)	481 (24.58%)	430 (23.49%)	420 (24.54%)	385 (22.70%)	
Some college or AA degree	4,130 (32.57%)	1,883 (32.34%)	684 (37.59%)	566 (33.17%)	491 (30.28%)	506 (28.65%)	
College graduate or above	2,223 (21.19%)	599 (12.06%)	355 (23.33%)	389 (27.47%)	385 (30.30%)	495 (35.37%)	
**Marital status (** ***n*** **, %)**							
Married	6,678 (52.43%)	2,343 (39.94%)	920 (48.65%)	1,118 (64.78%)	1,113 (70.98%)	1,184 (69.8)	< 0.001
Widowed	1,025 (5.46%)	323 (3.93%)	96 (3.02%)	152 (5.47%)	179 (7.06%)	275 (12.55%)	
Divorced	1,904 (12.89%)	968 (14.29%)	248 (11.96%)	258 (12.44%)	231 (12.04%)	199 (10.32%)	
Separated	532 (2.92%)	330 (4.24%)	72 (2.19%)	58 (2.48%)	44 (1.67%)	28 (0.82%)	
Never married	2,151 (15.83%)	1,440 (22.40%)	385 (21.03%)	164 (8.72%)	95 (5.35%)	67 (3.99%)	
Living with partner	1,361 (10.46%)	917 (15.18%)	244 (13.14%)	111 (6.11%)	45 (2.91%)	44 (2.47%)	
**Diabetes (** ***n*** **, %)**							
No	11,401 (87.30%)	5,607 (91.27%)	1,689 (88.71%)	1,466 (84.46%)	1,272 (80.98%)	1,367 (80.69%)	< 0.001
Borderline	354 (2.31%)	122 (1.68%)	47 (2.59%)	64 (2.67%)	70 (3.44%)	51 (2.72%)	
Yes	1,896 (10.38%)	592 (7.05%)	229 (8.71%)	331 (12.87%)	365 (15.58%)	379 (16.58%)	
**Failing kidneys (** ***n*** **, %)**							
No	13,150 (97.17%)	6,161 (97.84%)	1,908 (97.80%)	1,780 (97.14%)	1,621 (96.04%)	1,680 (95.07%)	< 0.001
Yes	501 (2.83%)	160 (2.16%)	57 (2.20%)	81 (2.86%)	86 (3.96%)	117 (4.93%)	
**Heart failure (** ***n*** **, %)**							
No	13,100 (97.03%)	6,140 (97.79%)	1,887 (97.25%)	1,752 (95.88%)	1,632 (97.05%)	1,689 (95.32%)	< 0.001
Yes	551 (2.97%)	181 (2.21%)	78 (2.75%)	109 (4.12%)	75 (2.95%)	108 (4.68%)	
**Coronary heart disease (** ***n*** **, %)**							
No	12,921 (95.42%)	6,112 (97.01%)	1,872 (96.53%)	1,741 (94.67%)	1,580 (93.49%)	1,616 (91.02%)	< 0.001
Yes	730 (4.58%)	209 (2.99%)	93 (3.47%)	120 (5.33%)	127 (6.51%)	181 (8.98%)	
**Emphysema (** ***n*** **, %)**							
No	13,083 (96.37%)	6,045 (96.00%)	1,890 (96.71%)	1,761 (95.73%)	1,642 (97.03%)	1,745 (97.38%)	0.130
Yes	568 (3.63%)	276 (4.00%)	75 (3.29%)	100 (4.27%)	65 (2.97%)	52 (2.62%)	
**Chronic bronchitis (** ***n*** **, %)**							
No	12,478 (91.41%)	5,699 (89.70%)	1,812 (93.17%)	1,709 (92.00%)	1,582 (92.69%)	1,676 (93.46%)	< 0.001
Yes	1,173 (8.59%)	622 (10.30%)	153 (6.83%)	152 (8.00%)	125 (7.31%)	121 (6.54%)	
**Cancer or malignancy (** ***n*** **, %)**							
No	12,090 (88.15%)	5,861 (92.01%)	1,826 (93.10%)	1,631 (88.12%)	1,414 (81.82%)	1,358 (74.10%)	< 0.001
Yes	1,561 (11.85%)	460 (7.99%)	139 (6.90%)	230 (11.88%)	293 (18.18%)	439 (25.90%)	
**Depression (** ***n*** **, %)**							
Yes	1,560 (10.35%)	982 (14.69%)	206 (8.37%)	152 (6.30%)	126 (6.24%)	94 (5.90%)	
No	12,091 (89.65%)	5,339 (85.31%)	1,759 (91.63%)	1,709 (93.70%)	1,581 (93.76%)	1,703 (94.10%)	

Continuous variables are mean and variance, and categorical variables are proportions and confidence intervals.

Q0, not quit smoking (0 days); Q1, 1st quartile of smoking cessation duration (< 5 years); Q2, 2nd quartile of smoking cessation duration (5–15 years); Q3, 3rd quartile of smoking cessation duration (15–29 years); Q4, 4th quartile of smoking cessation duration (>29 years).

In addition, we observed differences (*P* < 0.05) in age, BMI, gender, race, education level, marital status, and family PIR between depressed and non-depressed individuals (Table 8). Diabetes, renal failure, heart failure, coronary heart disease, chronic bronchitis, emphysema, nausea, and tumor were highly correlated with depression (*P* < 0.05) (Table 8). Compared with the other four groups, the Q4 group was the least prone to depression (OR = 0.36, 95% CI: 0.27–0.48, *P* < 0.001).

**Table 8:** Univariate analysis of depressive symptoms in the study population based on smoking cessation status.

**Characteristics**	**Statistics**	**OR, 95%CI, *P*-value**
Age, year(mean, se)	48.65 ± 0.27	0.99 (0.99–1.00) < 0.001
BMI, kg/m^2^ (mean, se)	29.06 ± 0.09	1.02 (1.01–1.03) < 0.001
Family PIR (mean, se)	2.84 ± 0.03	0.67 (0.63–0.71) < 0.001
**Gender (** ***n*** **, %)**		
Male	8,019 (55.80%)	1
Female	5,632 (44.20%)	2.08 (1.83–2.36) < 0.001
**Race (** ***n*** **, %)**		
Mexican American	1,691 (6.30%)	1
Other Hispanic	1,080 (4.18%)	1.57 (1.17–2.11) 0.003
Non-Hispanic white	7,028 (74.12%)	1.06 (0.86–1.30) 0.609
Non-Hispanic black	2,820 (9.55%)	1.49 (1.21–1.84) < 0.001
Other races	1,032 (5.84%)	1.37 (1.05–1.78) 0.021
**Education level (** ***n*** **, %)**		
< 9th grade	1,260 (4.95%)	1
9–11th grade	2,417 (13.80%)	1.00 (0.78–1.26) 0.972
High school graduate	3,621 (27.49%)	0.69 (0.55–0.87) 0.002
Some college or AA degree	4,130 (32.57%)	0.70 (0.55–0.88) 0.003
College graduate or above	2,223 (21.19%)	0.30 (0.20–0.44) < 0.001
**Marital status (** ***n*** **, %)**		
Married	6,678 (52.43%)	1
Widowed	1,025 (5.46%)	1.76 (1.30–2.37) < 0.001
Divorced	1,904 (12.89%)	2.46 (2.06–2.93) < 0.001
Separated	532 (2.92%)	3.71 (2.96–4.65) < 0.001
Never married	2,151 (15.83%)	1.87 (1.55–2.27) < 0.001
Living with partner	1,361 (10.46%)	1.85 (1.50–2.29) < 0.001
**Diabetes (** ***n*** **, %)**		
No	11,401 (87.30%)	1
Borderline	354 (2.31%)	1.37 (0.96–1.96) 0.090
Yes	1,896 (10.38%)	1.58 (1.33–1.87) < 0.001
**Failing kidneys (** ***n*** **, %)**		
No	13,150 (97.17%)	1
Yes	501 (2.83%)	2.65 (2.05–3.42) < 0.001
**Heart failure (** ***n*** **, %)**		
No	13,100 (97.03%)	1
Yes	551 (2.97%)	2.25 (1.72–2.95) < 0.001
**Coronary heart disease (** ***n*** **, %)**		
No	12,921 (95.42%)	1
Yes	730 (4.58%)	1.32 (0.99–1.77) 0.062
**Emphysema (** ***n*** **, %)**		
No	13,083 (96.37%)	1
Yes	568 (3.63%)	2.33 (1.82–2.98) < 0.001
**Chronic bronchitis (** ***n*** **, %)**		
No	12,478 (91.41%)	1
Yes	1,173 (8.59%)	2.90 (2.41–3.48) < 0.001
**Cancer or malignancy (** ***n*** **, %)**		
No	12,090 (88.15%)	1
Yes	1,561 (11.85%)	1.11 (0.90–1.37) 0.321
**Smoking cessation duration**		
Q0	6,321 (46.3%)	1
Q1	1,965 (14.4%)	0.53 (0.44–0.64) < 0.001
Q2	1,861 (13.6%)	0.39 (0.30–0.51) < 0.001
Q3	1,707 (12.5%)	0.39 (0.29–0.51) < 0.001
Q4	1,797 (13.2%)	0.36 (0.27–0.48) < 0.001

CI, confidence interval; OR, odds ratio.

Continuous variables are mean and variance, and categorical variables are proportions and confidence intervals.

Q0, not quit smoking (0 days); Q1, 1st quartile of smoking cessation duration (< 5 years); Q2, 2nd quartile of smoking cessation duration (5–15 years); Q3, 3rd quartile of smoking cessation duration (15–29 years); Q4, 4th quartile of smoking cessation duration (>29 years).

**Figure 3:**
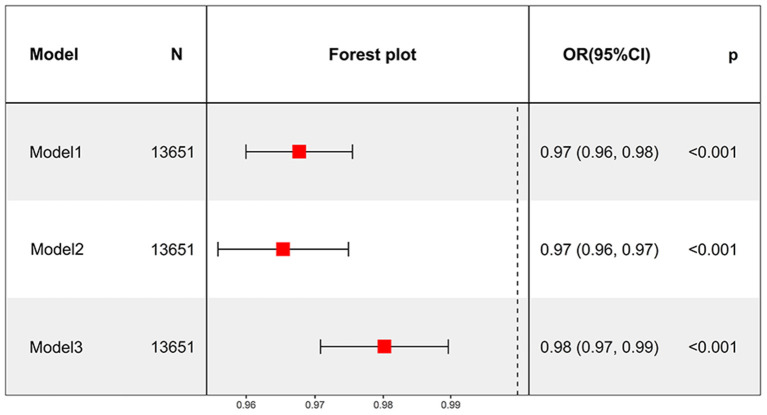
Smoking cessation duration as a continuous variable to analyze the relationship between smoking cessation and depression.

Model III confirmed that each additional year of smoking cessation was associated with a 2% decrease in the likelihood of developing depression after adjusting for most confounders (OR = 0.98, 95% CI: 0.97–0.99, *P* < 0.001) (Figure 3). After converting continuous variables to categorical variables, all three models still showed that smoking cessation duration was inversely related to depression (all models, *P* for trend < 0.001). Model III revealed a 45% reduced risk of depression in both the Q3 and Q4 groups (Table 9).

**Table 9:** Smoking cessation duration as categorical variables to analyze the relationship between smoking cessation and depression in adults (*n* = 13,651) in NHANES (2005–2018).

	**OR (95%CI)**, ***P*****-value**		
	**Model 1**	**Model 2**	**Model 3**
**Smoking cessation duration**			
Q0	1	1	1
Q1	0.53 (0.44–0.64) < 0.001	0.52 (0.42–0.63) < 0.001	0.63 (0.51–0.78) < 0.001
Q2	0.39 (0.30–0.51) < 0.001	0.37 (0.27–0.49) < 0.001	0.48 (0.36–0.65) < 0.001
Q3	0.39 (0.29–0.51) < 0.001	0.36 (0.27–0.48) < 0.001	0.55 (0.40–0.75) < 0.001
Q4	0.36 (0.27–0.48) < 0.001	0.35 (0.25–0.49) < 0.001	0.55 (0.39–0.79) < 0.001
*P* for trend	< 0.001	< 0.001	< 0.001

Model 1: adjust for none. Model 2, adjust for age, gender, BMI, race. Model 3, adjust for age, gender, BMI, race, educational level, marital status, family PIR, diabetes, failing kidneys, heart failure, coronary heart disease, emphysema, chronic bronchitis, cancer or malignancy.

P for trend was calculated by modeling the median value of quit smoking time in each quartile as a continuous variable.

CI, confidence interval; OR, odds ratio.

Q0, not quit smoking (0 days); Q1, 1st quartile of smoking cessation duration (< 5 years); Q2, 2nd quartile of smoking cessation duration (5–15 years); Q3, 3rd quartile of smoking cessation duration (15–29 years); Q4, 4th quartile of smoking cessation duration (>29 years).

## 4. Discussion

There is ongoing controversy regarding whether smoking increases the risk of depression (6, 15) or not (16–19). The present study first examined the association between smoking status and depression by analyzing data in the NHANES database (2005–2018). It was found that smokers had a significantly increased likelihood of developing depression than non-smokers. Compared with people who have never been exposed to tobacco (never smokers), those who have smoked in the past (previous smokers), currently smoke (occasional smokers), and who smoke every day (daily smokers) had an increased risk of depression in the given order. This result supports a conclusion that has been increasingly accepted in recent years: smoking increases the risk of depression (20). Next, we analyzed the relationship between smoking volume and depression. The findings showed that the higher the daily smoking volume, the more severe the risk of depression. This result confirms that smoking volume is positively related to the risk of depression, which is similar to the findings of some previous studies (21), and further supports the conclusion that smoking increases the risk of depression. These associations persisted even after adjusting for known confounders such as age, gender, BMI, race, education level, marital status, family PIR, diabetes, failing kidneys, heart failure, coronary heart disease, emphysema, chronic bronchitis, and cancer or malignancy.

However, some scholars believe that the high incidence of depression in smokers is not caused by smoking but that depressed people tend to smoke because they prefer to use tobacco to improve their mental status—the self-medication hypothesis (22, 23). Tobacco contains substances, such as nicotine, which are primarily used to excite the brain. Nicotine mainly acts on nicotinic acetylcholine receptors (nAChRs), which stimulate the release of substances such as norepinephrine, serotonin, dopamine, acetylcholine, γ-aminobutyric acid, and glutamate secretion in the brain (24). The brain regions involved in these activities include the lateral septum, dorsal raphe nucleus, mesolimbic dopamine system, and hippocampus, which regulate stress response, anxiety, and depression pathways, thus affecting anxiety level and mood (25). Although the short-term pharmacological effect of nicotine is to stimulate the brain and relieve stress, anxiety, and depression (26), long-term use of nicotine is prone to addiction, which can cause mental and psychological damage, making people more sensitive and vulnerable, and more prone to depression or anxiety (27). These explanations also corroborate the idea that smoking increases the risk of depression.

Finally, we analyzed the association between smoking cessation and depression, and the results showed that the longer the smoking cessation duration, the lower the risk of depression. This inversely proves that smoking increases the risk of depression. As nicotine is addictive, withdrawal symptoms early in smoking cessation can aggravate discomfort, causing anxiety and depression (28). If the self-medication hypothesis holds that smokers smoke to relieve mental discomfort, they will have difficulty quitting smoking. Moreover, smoking cessation is associated with an increased risk of depression due to the loss of the therapeutic effect of tobacco. This inference is contrary to the findings of this study and some previous similar studies (11). It has even been reported that smokers feel happier when they quit smoking, which researchers fear could be a reason for people to smoke (29, 30). However, according to our results, although former smokers had a reduced risk of depression compared with smokers, they had a higher risk of depression than those who had never been exposed to nicotine, which may be due to the toxic effects of long-term nicotine use on the nervous system (4). Thus, smoking for the joy of quitting smoking could lead to adverse health outcomes.

This study has several strengths: (1) The data are obtained from the NHANES database, which is relatively standardized and increases the reliability of the results; (2) Cross-sectional research with a large sample size, standardized data collection, and reliable information is more objective (31); (3) We analyzed the relationship between smoking and depression from three aspects, that is, smoking status, smoking volume, and smoking cessation. The study also has shortcomings. First, the data we analyzed came from US citizens; therefore, the results of this study may not be applicable to other countries or regions. Second, this is a cross-sectional study, and the evidence of causality is not as good as in longitudinal studies, let alone clinical randomized control analysis.

## 5. Conclusions

In summary, we believe that smoking is a behavior that increases the risk of depression. The higher the smoking frequency and smoking volume, the higher the risk of depression, whereas smoking cessation reduces the risk of depression, and the longer the smoking cessation duration, the lower the risk of depression.

## Data availability statement

The original contributions presented in the study are included in the article, further inquiries can be directed to the corresponding author.

## Ethics statement

The studies involving human participants were reviewed and approved by the Research Ethics Review Board of the National Center for Health Statistics. The patients/participants provided their written informed consent to participate in this study.

## Author contributions

ZW and XZ reviewed the literature, analyzed the data, and wrote the manuscript. ZZha, JW, YY, and QY reviewed the literature. ZZho, LT, and JY prepared all the figures and tables. All authors read and approved the final manuscript.
